# Lafayette Ranney, M.D.

**Published:** 1883-03

**Authors:** 


Dr. Lafayette Ranney, a well-known physician in this city, died of pneumonia at his residence, No. 14 West Thirty-second Street, Thursday, February 15th. Dr. Ranney was a member of an old and distinguished Vermont family, and his father was for many years Lieutenant Governor of that State. He was born in 1819, and was educated at Dartmouth College, graduating in 1845. Shortly after he came to this city to practice. Dr. Ranney's career in this city was one of considerable distinction. He was for many years a Commissioner of Education, and was one of the leading members of the Broadway Tabernacle Church. Of late he had led a life of great retirement. Dr. Ranney was twice married. He leaves four sons, two of whom are physicians, the eldest, Dr. Ambrose L. Ranney, being adjunct professor of anatomy in the University Medical College.

